# PACAP is Implicated in the Stress Axes

**DOI:** 10.2174/138161211795589382

**Published:** 2011-04

**Authors:** Hitoshi Hashimoto, Norihito Shintani, Mamoru Tanida, Atsuko Hayata, Ryota Hashimoto, Akemichi Baba

**Affiliations:** 1 Laboratory of Molecular Neuropharmacology, Graduate School of Pharmaceutical Sciences, Osaka University, 1-6 Yamadaoka, Suita, Osaka 565-0871, Japan; 2 Center for Child Mental Development, United Graduate School of Child Development, Osaka University, Kanazawa University and Hamamatsu University School of Medicine, 2-2 Yamadaoka, Suita, Osaka 565-0871, Japan; 3 Department of Molecular Pharmaceutical Science, Osaka University Graduate School of Medicine, 2-2 Yamadaoka, Suita, Osaka 565-0871, Japan; 4 Department of Biomedical Sciences, College of Life Sciences, Ritsumeikan University, 1-1-1 Nojihigashi, Kusatsu, Shiga 525-8577, Japan; 5 Department of Psychiatry, Osaka University Graduate School of Medicine, 2-2 Yamadaoka, Suita, Osaka 565-0871, Japan; 6 Hyogo University of Health Sciences, 1-3-6 Minatojima, Chuo-ku, Kobe, Hyogo 650-8530, Japan

**Keywords:** Hypothalamic-pituitary-adrenal (HPA) axis, PACAP, paraventricular nucleus (PVN) of the hypothalamus, post-traumatic disorder (PTSD), psychiatric disorders, stress, sympathoadrenomedullary system, sympathetic nervous system.

## Abstract

Pituitary adenylate cyclase-activating polypeptide (PACAP) is a highly conserved pleiotropic neuropeptide that functions as a neurotransmitter, neuromodulator and neurotrophic factor. Accumulating evidence implicates PACAP as an important regulator of both central and/or peripheral components of the stress axes, particularly exposure to prolonged or traumatic stress. Indeed, PACAP and its cognate receptors are widely expressed in the brain regions and peripheral tissues that mediate stress-related responses. In the sympathoadrenomedullary system, PACAP is required for sustained epinephrine secretion during metabolic stress. It is likely that PACAP regulates autonomic function and contributes to peripheral homeostasis by maintaining a balance between sympathetic and parasympathetic activity, favoring stimulation of the sympathetic system. Furthermore, PACAP is thought to act centrally on the paraventricular nucleus of the hypothalamus to regulate both the hypothalamic-pituitary-adrenal axis and the sympathetic nervous system. Intriguingly, PACAP is also active in brain structures that mediate anxiety- and fear-related behaviors, and the expression of PACAP and its receptors are dynamically altered under pathologic conditions. Thus PACAP may influence both hard-wired (genetically determined) stress responses and gene-environment interactions in stress-related psychopathology. This article aims to overview the molecular mechanisms and psychiatric implications of PACAP-dependent stress responses.

## INTRODUCTION

Stress is defined as a state of threat, or perceived threat to homeostasis and can be classified under four main categories: 1) physical stressors; 2) psychological stressors that reflect a learned response to previously experienced adverse conditions; 3) social stressors reflecting disturbed interactions among individuals; and 4) stressors that challenge cardiovascular and metabolic homeostasis [1, 2]. Adaptation to stressful stimuli is a priority for all organisms and involves the activation of specific central circuits. Adaptive responses are genetically and constitutionally programmed (“hard-wired”) and are constantly modulated by environmental factors (“gene-environmental interaction”). Stress axes comprising the sympathoadrenal and sympathoneuronal systems and the hypothalamic-pituitary-adrenocortical axis, respond to different types of stressors and exhibit stressor-specific response patterns [1-3].

Neuropeptides tend to exert a long-lasting modulatory effect on the small-molecule neurotransmitters with which they colocalize, by regulating the response times of second messenger systems [4]. Thus, neuropeptides are important for adaptation of the nervous system to pathophysiological conditions. The neuropeptide, pituitary adenylate cyclase-activating polypeptide (PACAP), has long been recognized as an important regulator of central and peripheral components of the stress axes, as outlined in Table **1**.

## PITUITARY ADENYLATE CYCLASE-ACTIVATING POLYPEPTIDE (PACAP)

PACAP was first identified as a novel hypothalamic neuropeptide in 1989 by Miyata *et al*., based on its ability to stimulate adenylate cyclase in rat anterior pituitary cell cultures [5]. PACAP exists in two biologically active forms, PACAP-38 and the C-terminally truncated PACAP-27. PACAP-27 has an amino acid sequence 68% consistent with vasoactive intestinal polypeptide (VIP) and 37% with secretin, indicating that PACAP is a member of the VIP/glucagon/growth hormone-releasing hormone (GHRH)/secretin superfamily [6].

The primary structure of PACAP-38 is completely conserved across all mammalian species studied to date, and is virtually unchanged between mammals, lower vertebrates and protochordates [7]. This structural conservation is a testimony to the vital physiological role of PACAP. PACAP and the three subtypes of PACAP cognate receptors, PAC_1_, VPAC_1_ and VPAC_2_, are found in both the central nervous system and the periphery. In the central nervous system, PACAP is involved in a variety of physiological and pathophysiological processes, including those underlying higher brain function, emotional and behavioral regulation and neuroprotection [6].

## DISTRIBUTION OF PACAP AND ITS RECEPTORS IN STRESS-RELATED NEURAL SYSTEMS

Stress responses are mediated by specialized neural circuits in the limbic forebrain, hypothalamus and brainstem, which activate and control neuroendocrine and autonomic stress systems [1-3].

In the rat, PACAP is most abundant in the hypothalamic area. Indeed, PACAP-immunoreactive neurons and PACAP receptors are primarily located in the magnocellular region of the paraventricular nucleus (PVN) of the hypothalamus and in the supraoptic nucleus of the hypothalamus, structures which produce the stress-related hormones, oxytocin and vasopressin [6, 8-10]. Dense PACAP-immunoreactive fibers can be observed in the median eminence and in the hypothalamo-hypophysial portal system [6, 8, 9], and a higher amount of PACAP has also been detected in hypophysial portal blood compared with peripheral blood [11].

PACAP and PACAP receptors are also widely distributed in brain areas outside the hypothalamo-hypophysis known to be involved in stress responses, including the medial prefrontal cortex, hippocampus, amygdala, bed nucleus of the stria terminalis (BNST) and adrenal gland [6, 8, 12-14].

PAC_1_ receptor mRNA is generally the most widely and abundantly expressed of the three PACAP receptor subtypes, although there are exceptions, for example VPAC_1_ receptor mRNA is abundantly expressed in hippocampal pyramidal neurons [15] and VPAC_2_ receptor mRNA is abundantly expressed in the central amygdala [16].

At a subcellular level, PACAP-immunoreactivity appears to be concentrated within neuronal cell bodies and dendrites. Ultrastructurally, it is noteworthy that immunostaining for the PAC_1_ receptor revealed its presence on synaptic membranes [13].

Detailed anatomical information regarding stress responses are discussed in the following sections.

## PACAP-DEPENDENT STIMULATION OF THE SYMPATHOADRENOMEDULLARY SYSTEM

Many studies have focused on the role of PACAP in the sympathoadrenomedullary system. Retrograde tracing studies with the cholera toxin B subunit have shown that PACAP is expressed in various hypothalamic and extrahypothalamic regions, and in the catecholaminergic neurons of the brain stem that innervate the PVN and the intermediolateral cell column of the spinal cord [17, 18]. Additionally, sympathetic preganglionic neurons projecting to the adrenal medulla contain PACAP [19, 20].

PACAP is postulated to function as an "emergency response" cotransmitter in the sympathoadrenal axis during metabolic stress, by ensuring sustained secretory responses [21]. During insulin-induced hypoglycemia, PACAP-deficient mice exhibit impaired long-term secretion of epinephrine due to defective compensatory stimulation of catecholamine biosynthesis [21]. In addition, adaptive thermogenesis is impaired in PACAP-deficient mice subjected to prolonged periods of cold stress, due to insufficient norepinephrine stimulation of brown adipose tissue [22]. Furthermore, synthesis of adrenaline-synthesizing enzymes in the adrenal medulla is decreased in PACAP-deficient mice subjected to a psychological stressor such as restraint stress, suggesting that PACAP is a key neuroendocrine factor in the hypothalamus and adrenal gland [23]. In adrenal chromaffin cells, PACAP exerts a stimulatory effect on catecholamine secretion, which is associated with increased expression of catecholamine biosynthetic enzyme genes [24].

## PACAP-DEPENDENT STIMULATION OF OTHER SYMPATHETIC PATHWAYS

The demonstration of preproPACAP within the sympathetic preganglionic neurons that innervate the adrenal gland, and in those at level C8-T10 of the spinal cord, suggests that PACAP is widely distributed in the sympathetic nervous system [20]. PAC_1_ receptor mRNA is intensely expressed in virtually all principal neurons of the sympathetic superior cervical ganglia in rat, and PACAP mRNA is expressed with variable intensity in approximately half of the principal neurons in this structure [25].

*In vivo* electrophysiological studies have shown that PACAP injected into the third ventricle activates sympathetic fibers that innervate brown adipose tissue, the kidneys, adrenal gland, and other abdominal viscera, whereas it suppresses parasympathetic nerve activities [26]. These results suggest that PACAP regulates autonomic function and contributes to peripheral homeostasis by maintaining a balance between sympathetic and parasympathetic activity [26].

## PACAP-DEPENDENT STIMULATION OF THE HYPOTHALAMICPITUITARY-ADRENAL (HPA) SYSTEM

Immunohistochemical studies have revealed that a significant proportion of PACAP-immunoreactive neurons in the PVN co-express corticotropin-releasing factor (CRF) [9]. Electron microscopic studies have revealed the presence of synapses between PACAP-containing terminals and CRF-perikarya and -dendrites [27]. PACAP injected intracerebroventricularly activates CRF gene expression in the rat PVN [28], while intravenously infused PACAP induces a dose-dependent increase in serum adrenocorticotropin levels in human [29]. In vitro, PACAP increases adrenocorticotropin release from superfused rat pituitary cells [5] and the pro-opiomelanocortin gene expression in the mouse corticotrope-derived cell line AtT20 [30, 31]. Intracerebroventricular and intracisternal injection of PACAP also increases plasma vasopressin secretion [32, 33]. PACAP is localized in nerve terminals that innervate vasopressin-containing neurons, and PAC1 receptor mRNA is highly expressed in vasopressin-containing neurons in the rat hypothalamic supraoptic nucleus [10]. In the posterior pituitary, PACAP stimulates the release of vasopressin through the cAMP signalling pathway [34]. Together, these data provide strong evidence that PACAP is involved in the activation of the HPA axis.

PACAP-deficient mice show a decrease in corticosterone secretion in response to trimethyltin exposure [35], restraint stress [23], or light following constant darkness [36]. Corticosterone secretion elicited by restraint stress is more severely impaired in PACAP-deficient mice, especially when the stress is prolonged [23]. The increase in hypothalamic mRNA expression of Egr1 (early growth response 1), c-Fos (FBJ osteosarcoma oncogene), and CRF seen with restraint stress, is much less pronounced in PACAP-deficient mice than in wild-type mice [23]. Taken together, these results suggest that PACAP plays an important role in the activation of hypothalamic neurons, in order to regulate the HPA axis response to stress.

## MECHANISTIC INSIGHTS INTO PACAP-DEPENDENT STRESS RESPONSES AND IMPLICATIONS FOR PSYCHIATRIC DISORDERS

It has been demonstrated that intracerebroventricular injection of PACAP induces acute stress-related behaviors in rodents (e.g. body grooming and wet-dog shakes), which are associated with an elevation in plasma corticosterone levels and activation of cAMP response element-mediated CRF gene transcription [37].

Intriguingly, chronic unpredictable stress dramatically increases mRNA expression of PACAP and brain-derived neurotrophic factor (BDNF) in the dorsal part of the BNST, a region of the central extended amygdala that mediates fear- and anxiety-like behavior. Moreover, intra-BNST infusion of PACAP induces an anxiogenic response [38]. These findings implicate PACAP in stress-induced increases in BNST neuroplasticity and in long-term stress-induced behavioral changes [39]. Since BDNF expression is stimulated by PACAP [40], and is decreased in PAC1 receptor-deficient mice [41], PACAP-stimulated BDNF expression may underlie the dorsolateral BNST cellular plasticity associated with anxiety and behavior disorders [38,39]. Infusion of PACAP into the central nucleus of the amygdala has also been shown to induce stress-related behaviors [42].

PACAP-deficient mice exhibit remarkable behavioral abnormalities, and can be regarded as an animal model of psychotic behavior and depression [43-46]. Genetic association studies have provided evidence that single nucleotide polymorphisms in the PACAP gene are associated with an increased risk for schizophrenia [47] or major depressive disorders [48]. In addition, PACAP increases expression of disrupted in schizophrenia 1 (DISC1), which is recognized as a leading candidate risk gene for schizophrenia, and markedly but transiently decreases the association between DISC1 and the DISC1-interacting protein DBZ [49]. These data suggest that PACAP signaling might contribute to the pathogenesis of psychiatric disorders.

It is widely accepted that inadequate response and/or prolonged exposure to stressors may result in psychiatric disorders [1-3]. It is therefore plausible that altered PACAP signaling systems may be involved in the etiology of stress-related psychiatric conditions. Maladaptive stress responses (either in hyperfunctional or hypofunctional states), and subsequent development of disease symptoms, involve an interplay of genetic factors and environmental adversity during critical periods characterized by increased vulnerability to stressors [1-3]. Indeed, PACAP-deficient mice housed alone for a two-week period from 4-weeks of age are more aggressive, but otherwise normal. Furthermore, correction of abnormal behavior by rearing mutant mice for 4 weeks in an enriched environment is effective when carried out from 4-weeks of age, but is ineffective when carried out from 8-weeks of age [50].

Finally, an important study by Ressler and colleagues [51] has shown a female-specific association of the PACAP–PAC1 receptor pathway with post-traumatic stress disorder (PTSD). Ressler *et al*. demonstrated that PTSD diagnosis was associated with peripheral blood levels of PACAP in females (higher levels in the PTSD cohort), and that an SNP in a putative estrogen response element of the PAC1 receptor gene, rs2267735, was associated with PTSD diagnosis, the severity of symptoms, and with PAC1 receptor messenger RNA expression in the brain. In this report, they also revealed that PACAP and PAC1 receptor mRNA levels were inversely correlated in the human cortex, suggesting that brain levels of PACAP peptide and PAC1 mRNA are tightly regulated [51]. In support, it has been shown that, in rat primary hippocampal neurons, selective serotonin reuptake inhibitors up-regulate PACAP expression and down-regulate PACAP receptor (PAC1 and VPAC2) expression, while the tricyclic antidepressant imipramine shows an opposite effect [52]. In addition, in mice chronically treated with phencyclidine, an N-methyl-D-aspartic acid receptor antagonist, PACAP mRNA is reduced, while PAC1 receptor mRNA is increased in the frontal cortex [47]. These data suggest that the expression of PACAP peptide and PAC1 receptors are actively regulated under physiologic and pathologic conditions, and that perturbations in the PACAP–PAC1 pathway are involved in abnormal stress responses underlying psychiatric conditions such as PTSD [51].

## CONCLUSIONS AND OUTLOOK

PACAP and its cognate receptors are widely expressed in brain regions and peripheral tissues known to be involved in the stress axes, and their expression levels may be dynamically altered under pathologic conditions. Convergent evidence implicates PACAP in stress-related responses, particularly during exposure to persistent stress. Under stressful conditions, PACAP coordinates stress signaling in the central and peripheral nervous system [23], and probably acts in concert with other neurotransmitters and neurotrophins. It is intriguing that PACAP also acts on brain structures that mediate anxiety- and fear-related behaviors, where it may influence both hard-wired (genetically determined) stress responses and gene-environment interactions in stress-related psychopathology.

The anxiogenic and/or fear-evoking properties of PACAP must be appropriate and controlled to enable development of a healthy stress response. Further study is required to establish how this neuropeptidergic system is involved in psychopathology under conditions of toxic stress (e.g. prolonged stress and emotional trauma). Future studies addressing the molecular basis and pathophysiological implications of PACAP-dependent stress responses may facilitate the development of drug therapies for stress.

## Figures and Tables

**Table 1:** PACAP-dependent Stress Responses

Effect or Implication of PACAP		References

BNST	Dramatic increase in PACAP and BDNF mRNA expression by chronic unpredictable stress Anxiogenic response induced by intra-BNST infusion of PACAP	[38, 39]

Amygdala	Stress-related behaviors induced by infusion of PACAP into the central nucleus of the amygdala	[42]

HPA axis	Stimulation of CRF gene expression in the PVN	[9, 23, 28, 37]
	Stimulation of pro-opiomelanocortin gene expression and adrenocorticotropin release	[5, 29-31]
	Activation of vasopressin-containing neurons in the supraoptic nucleus and plasma vasopressin secretion	[10, 32-34]
	Impairment of stress-induced corticosterone secretion in PACAP-deficient mice	[23]
	Acute stress-related behaviors and increased plasma corticosterone induced by intracerebroventricular injection of PACAP	[37]

Sympathoadreno medullary system	Sustained epinephrine secretion during prolonged stress by enhancement of compensatory catecholamine synthesis	[21, 23, 24]

Automonic nervous system	Stimulation of sympathetic outflow Maintenance of a sympathetic–parasympathetic balance	[26]

PTSD	Female-specific association of the PACAP–PAC1 receptor pathway with PTSD	[51]

BDNF: brain-derived neurotrophic factor BNST: bed nucleus of the stria terminalis HPA axis: hypothalamic-pituitary-adrenal axis PTSD: post-traumatic stress disorder PVN: paraventricular nucleus of the hypothalamus

## References

[1] Pacak K, Palkovits M (2001). Stressor specificity of central neuroendocrine responses: implications for stress-related disorders. Endocr Rev.

[2] Ulrich-Lai YM, Herman JP (2009). Neural regulation of endocrine and autonomic stress responses. Nat Rev Neurosci.

[3] Charmandari E, Tsigos C, Chrousos G (2005). Endocrinology of the stress response. Annu Rev Physiol.

[4] Hashimoto H, Shintani N, Baba A (2002). Higher brain functions of PACAP and a homologous Drosophila memory gene amnesiac: insights from knockouts and mutants. Biochem Biophys Res Commun.

[5] Miyata A, Arimura A, Dahl RR (1989). Isolation of a novel 38 residue-hypothalamic polypeptide which stimulates adenylate cyclase in pituitary cells. Biochem Biophys Res Commun.

[6] Vaudry D, Falluel-Morel A, Bourgault S (2009). Pituitary adenylate cyclase-activating polypeptide and its receptors: 20 years after the discovery. Pharmacol Rev.

[7] Sherwood NM, Krueckl SL, McRory JE (2000). The origin and function of the pituitary adenylate cyclase-activating polypeptide (PACAP)/glucagon superfamily. Endocr Rev.

[8] Koves K, Arimura A, Gorcs TG, Somogyvari-Vigh A (1991). Comparative distribution of immunoreactive pituitary adenylate cyclase activating polypeptide and vasoactive intestinal polypeptide in rat forebrain. Neuroendocrinology.

[9] Hannibal J, Mikkelsen JD, Fahrenkrug J, Larsen PJ (1995). Pituitary adenylate cyclase-activating peptide gene expression in corticotropin-releasing factor-containing parvicellular neurons of the rat hypothalamic paraventricular nucleus is induced by colchicine, but not by adrenalectomy, acute osmotic, ether, or restraint stress. Endocrinology.

[10] Shioda S, Yada T, Nakajo S, Nakaya K, Nakai Y, Arimura A (1997). Pituitary adenylate cyclase-activating polypeptide (PACAP): a novel regulator of vasopressin-containing neurons. Brain Res.

[11] Dow RC, Bennie J, Fink G (1994). Pituitary adenylate cyclase-activating peptide-38 (PACAP)-38 is released into hypophysial portal blood in the normal male and female rat. J Endocrinol.

[12] Hashimoto H, Nogi H, Mori K (1996). Distribution of the mRNA for a pituitary adenylate cyclase-activating polypeptide receptor in the rat brain: an in situ hybridization study. J Comp Neurol.

[13] Shioda S, Shuto Y, Somogyvari-Vigh A (1997). Localization and gene expression of the receptor for pituitary adenylate cyclase-activating polypeptide in the rat brain. Neurosci Res.

[14] Kozicz T, Vigh S, Arimura A (1997). Axon terminals containing PACAP- and VIP-immunoreactivity form synapses with CRF-immunoreactive neurons in the dorsolateral division of the bed nucleus of the stria terminalis in the rat. Brain Res.

[15] Ishihara T, Shigemoto R, Mori K, Takahashi K, Nagata S (1992). Functional expression and tissue distribution of a novel receptor for vasoactive intestinal polypeptide. Neuron.

[16] Usdin TB, Bonner TI, Mezey E (1994). Two receptors for vasoactive intestinal polypeptide with similar specificity and complementary distributions. Endocrinology.

[17] Das M, Vihlen CS, Legradi G (2007). Hypothalamic and brainstem sources of pituitary adenylate cyclase-activating polypeptide nerve fibers innervating the hypothalamic paraventricular nucleus in the rat. J Comp Neurol.

[18] Farnham MM, Li Q, Goodchild AK, Pilowsky PM (2008). PACAP is expressed in sympathoexcitatory bulbospinal C1 neurons of the brain stem and increases sympathetic nerve activity *in vivo*. Am J Physiol Regul Integr Comp Physiol.

[19] Holgert H, Holmberg K, Hannibal J (1996). PACAP in the adrenal
gland--relationship with choline acetyltransferase, enkephalin and
chromaffin cells and effects of immunological sympathectomy. Neuroreport.

[20] Kumar NN, Allen K, Parker L, Damanhuri H, Goodchild AK (2010). Neuropeptide coding of sympathetic preganglionic neurons, focus on adrenally projecting populations. Neuroscience.

[21] Hamelink C, Tjurmina O, Damadzic R (2002). Pituitary adenylate cyclase-activating polypeptide is a sympathoadrenal neurotransmitter involved in catecholamine regulation and glucohomeostasis. Proc Natl Acad Sci U S A.

[22] Gray SL, Yamaguchi N, Vencova P, Sherwood NM (2002). Temperature-sensitive phenotype in mice lacking pituitary adenylate cyclase-activating polypeptide. Endocrinology.

[23] Stroth N, Eiden LE (2010). Stress hormone synthesis in mouse hypothalamus and adrenal gland triggered by restraint is dependent on pituitary adenylate cyclase-activating polypeptide signaling. Neuroscience.

[24] Tonshoff C, Hemmick L, Evinger MJ (1997). Pituitary adenylate cyclase activating polypeptide (PACAP) regulates expression of catecholamine biosynthetic enzyme genes in bovine adrenal chromaffin cells. J Mol Neurosci.

[25] Nogi H, Hashimoto H, Hagihara N (1997). Distribution of mRNAs
for pituitary adenylate cyclase-activating polypeptide (PACAP),
PACAP receptor, vasoactive intestinal polypeptide (VIP), and VIP
receptors in the rat superior cervical ganglion. Neurosci Lett.

[26] Tanida M, Shintani N, Morita Y (2010). Regulation of autonomic nerve activities by central pituitary adenylate cyclase-activating polypeptide. Regul Pept.

[27] Legradi G, Hannibal J, Lechan RM (1998). Pituitary adenylate cyclase-activating polypeptide-nerve terminals densely innervate corticotropin-releasing hormone-neurons in the hypothalamic paraventricular nucleus of the rat. Neurosci Lett.

[28] Grinevich V, Fournier A, Pelletier G (1997). Effects of pituitary adenylate cyclase-activating polypeptide (PACAP) on corticotropin-releasing hormone (CRH) gene expression in the rat hypothalamic paraventricular nucleus. Brain Res.

[29] Chiodera P, Volpi R, Capretti L, Caffarri G, Magotti MG, Coiro V (1996). Effects of intravenously infused pituitary adenylate cyclase-activating polypeptide on adenohypophyseal Hormone secretion in normal men. Neuroendocrinology.

[30] Boutillier AL, Monnier D, Koch B, Loeffler JP (1994). Pituitary adenyl
cyclase-activating peptide: a hypophysiotropic factor that stimulates
proopiomelanocortin gene transcription, and proopiomelanocortin-
derived peptide secretion in corticotropic cells. Neuroendocrinology.

[31] Aoki Y, Iwasaki Y, Katahira M, Oiso Y, Saito H (1997). Regulation of the
rat proopiomelanocortin gene expression in AtT-20 cells. II: Effects
of the pituitary adenylate cyclase-activating polypeptide and
vasoactive intestinal polypeptide. Endocrinology.

[32] Murase T, Kondo K, Otake K, Oiso Y (1993). Pituitary adenylate cyclase-activating polypeptide stimulates arginine vasopressin release in conscious rats. Neuroendocrinology.

[33] Seki Y, Suzuki Y, Baskaya MK, Saito K, Takayasu M, Shibuya M, Sugita K (1995). Central cardiovascular effects induced by intracisternal PACAP in dogs. Am J Physiol.

[34] Lutz-Bucher B, Monnier D, Koch B (1996). Evidence for the presence of receptors for pituitary adenylate cyclase-activating polypeptide in the neurohypophysis that are positively coupled to cyclic AMP formation and neurohypophyseal hormone secretion. Neuroendocrinology.

[35] Morita Y, Yanagida D, Shintani N (2006). Lack of trimethyltin (TMT)-induced elevation of plasma corticosterone in PACAP-deficient mice. Ann N Y Acad Sci.

[36] Hatanaka M, Tanida M, Shintani N (2008). Lack of light-induced elevation of renal sympathetic nerve activity and plasma corticosterone levels in PACAP-deficient mice. Neurosci Lett.

[37] Agarwal A, Halvorson LM, Legradi G (2005). Pituitary adenylate cyclase-activating polypeptide (PACAP) mimics neuroendocrine and behavioral manifestations of stress: Evidence for PKA-mediated expression of the corticotropin-releasing hormone (CRH) gene. Brain Res Mol Brain Res.

[38] Hammack SE, Cheung J, Rhodes KM (2009). Chronic stress increases pituitary adenylate cyclase-activating peptide (PACAP) and brain-derived neurotrophic factor (BDNF) mRNA expression in the bed nucleus of the stria terminalis (BNST): roles for PACAP in anxiety-like behavior. Psychoneuroendocrinology.

[39] Hammack SE, Roman CW, Lezak KR (2010). Roles for pituitary adenylate cyclase-activating peptide (PACAP) expression and signaling in the bed nucleus of the stria terminalis (BNST) in mediating the behavioral consequences of chronic stress. J Mol Neurosci.

[40] Yaka R, He DY, Phamluong K, Ron D (2003). Pituitary adenylate cyclase-activating polypeptide (PACAP(1-38)) enhances N-methyl-D-aspartate receptor function and brain-derived neurotrophic factor expression via RACK1. J Biol Chem.

[41] Zink M, Otto C, Zorner B, Zacher C, Schutz G, Henn FA, Gass P (2004). Reduced expression of brain-derived neurotrophic factor in mice deficient for pituitary adenylate cyclase activating polypeptide type-I-receptor. Neurosci Lett.

[42] Legradi G, Das M, Giunta B, Hirani K, Mitchell EA, Diamond DM (2007). Microinfusion of pituitary adenylate cyclase-activating polypeptide into the central nucleus of amygdala of the rat produces a shift from an active to passive mode of coping in the shock-probe fear/defensive burying test. Neural Plast.

[43] Hashimoto H, Shintani N, Tanaka K (2001). Altered psychomotor behaviors in mice lacking pituitary adenylate cyclase-activating polypeptide (PACAP). Proc Natl Acad Sci U S A.

[44] Tanaka K, Shintani N, Hashimoto H (2006). Psychostimulant-induced attenuation of hyperactivity and prepulse inhibition deficits in Adcyap1-deficient mice. J Neurosci.

[45] Hashimoto H, Hashimoto R, Shintani N (2009). Depression-like behavior in the forced swimming test in PACAP-deficient mice: amelioration by the atypical antipsychotic risperidone. J Neurochem.

[46] Yamada K, Matsuzaki S, Hattori T (2010). Increased stathmin1 expression in the dentate gyrus of mice causes abnormal axonal arborizations. PLoS One.

[47] Hashimoto R, Hashimoto H, Shintani N (2007). Pituitary adenylate cyclase-activating polypeptide is associated with schizophrenia. Mol Psychiatry.

[48] Hashimoto R, Hashimoto H, Shintani N (2010). Possible association between the pituitary adenylate cyclase-activating polypeptide (PACAP) gene and major depressive disorder. Neurosci Lett.

[49] Hattori T, Baba K, Matsuzaki S (2007). A novel DISC1-interacting partner DISC1-Binding Zinc-finger protein: implication in the modulation of DISC1-dependent neurite outgrowth. Mol Psychiatry.

[50] Ishihama T, Ago Y, Shintani N (2010). Environmental factors during early developmental period influence psychobehavioral abnormalities in adult PACAP-deficient mice. Behav Brain Res.

[51] Ressler KJ, Mercer KB, Bradley B (2011). Post-traumatic stress disorder is associated with PACAP and the PAC1 receptor. Nature.

[52] Reichenstein M, Rehavi M, Pinhasov A (2008). Involvement of pituitary adenylate cyclase activating polypeptide (PACAP) and its receptors in the mechanism of antidepressant action. J Mol Neurosci.

